# Potassium and Phosphorus Fertilizer Impacts on Alfalfa Taproot Carbon and Nitrogen Reserve Accumulation and Use During Fall Acclimation and Initial Growth in Spring

**DOI:** 10.3389/fpls.2021.715936

**Published:** 2021-08-16

**Authors:** W. Kess Berg, Sylvie M. Brouder, Suzanne M. Cunningham, Jeffrey J. Volenec

**Affiliations:** Department of Agronomy, Purdue University, West Lafayette, IN, United States

**Keywords:** winter hardiness, plant nutrition, *Medicago sativa*, starch, amino acids, protein, sugars, cold acclimation

## Abstract

Phosphorus (P) and potassium (K) impact alfalfa (*Medicago sativa* L.) performance, but how these nutrients alter taproot physiology during fall acclimation and subsequent growth in spring is unclear. Our objectives were to: (1) determine seasonal patterns for taproot P and K concentrations during fall acclimation and during initial shoot growth in spring; (2) determine how P and K nutrition impacts accumulation of taproot C and N reserves during fall and their subsequent use when shoot growth resumes in spring; and (3) assess how addition of P and K fertilizer impacts survival and shoot growth in spring. Two P (0 and 75 kg ha^−1^) and two K (0 and 400 kg ha^−1^) treatments were applied and taproots were sampled between September and December, and again from March to May over 2 years. Concentrations of taproot sugar, starch, buffer-soluble protein, amino-N, and RNA pools were determined. While P and K fertilizer application increased taproot P and K concentrations two- to three-fold, concentrations of P and K in taproots over time did not change markedly during cold acclimation in fall, however, taproot P declined in spring as plant growth resumed. Compared to the 0K-0P treatment, taproots of plants fertilized with 400K-75P had higher starch, protein, amino-N, and RNA, but reduced sugar concentrations in fall. Concentrations of all these pools, except starch, declined during the initial 2 weeks of sampling beginning in late March as shoot growth resumed in spring. Herbage yield in May was highest for the 400K-75P treatment and least for the 0K-0P treatment, differences that were associated with variation in mass shoot^−1^ and not shoots m^−2^. High yield of the 400K-75P plants in May was consistently associated with greater concentrations and use of amino-N, soluble protein, and RNA pools in taproots, and not with accumulation and use of starch and sugar pools. Understanding factors leading to the accumulation of taproot N reserves and RNA during cold acclimation in fall and their use during the initial growth in spring should enhance efforts to improve alfalfa growth and herbage yield in spring.

## Introduction

Total nonstructural carbohydrates (sugar and starch) have long been positively associated with improved winter survival of alfalfa (Graber et al., 1927; Bula et al., 1956). More recently, the underlying physiological basis for improved winter survival and rapid shoot growth of alfalfa after harvest has been expanded to include taproot N reserves, including protein and amino acid pools (Hendershot and Volenec, 1993; Avice et al., 1996; Barber et al., 1996; Cunningham and Volenec, 1996; Volenec et al., 1996; Berg et al., 2009). Adequate P and K nutrition also has a positive impact on alfalfa yield and persistence (Berg et al., 2005, 2007; Lissbrant et al., 2010). While low K nutrition has traditionally been associated with poor persistence (Brown, 1928), application of fertilizer P to K-deficient soils can intensify plant losses (Berg et al., 2018; Volenec et al., 2021). The impact of P and K nutrition on accumulation of taproot C and N pools during fall and their subsequent utilization throughout winter and in early spring when growth resumes have not been critically evaluated. This information would enhance our understanding of the physiological mechanisms influenced by P and K that are associated with enhanced winter hardiness, improved plant persistence, and increased shoot growth in spring.

Accumulation of nonstructural carbohydrates, and especially soluble sugars, in alfalfa taproots in fall is thought to enhance tolerance to low temperatures and other stresses associated with winter (Castonguay et al., 2006). For example, fall-dormant, winter-hardy alfalfa cultivars had higher taproot sugar (not starch) concentrations when compared to non-dormant plants that died over winter (Cunningham and Volenec, 1998; Cunningham et al., 2001, 2003; Lu et al., 2018). These sugars have both a cryoprotective role, but also serve as respiratory substrates during winter and immediately after defoliation in summer when carbohydrates from photosynthesis are limited. Avice et al. (1996) used stable isotope labeling to demonstrate that nonstructural carbohydrate depletion from taproots of defoliated alfalfa was almost exclusively due to respiratory metabolism; very little of the ^13^C label was transferred from taproots to shoots during regrowth. However, high taproot sugar concentrations do not ensure winter hardiness. Using alfalfa cultivars differing in fall dormancy, Haagenson et al. (2003b) reported that the reduced winter survival of plants defoliated in mid-fall occurred despite these plants having higher taproot sugar concentrations when compared to plants left uncut in fall. Surprisingly, defoliation in mid-fall increased sugar concentrations 5-fold in taproots of the least fall dormant cultivar (fall dormancy = 9) to levels similar to those observed in uncut, moderately winter hardy, semi-dormant cultivars, but this increase was not associated with enhanced winter survival. These authors reported that changes in taproot protein concentrations were associated with cultivar- and defoliation-induced differences in winter survival.

Hendershot and Volenec (1993) showed that alfalfa taproots accumulate N as amino acids and protein during fall that are subsequently depleted when shoot growth resumes in spring, but did not relate these changes to agronomic performance. Later work (Li et al., 1996) confirmed fall accumulation/spring depletion of these N pools in taproots of several forage legumes, including birdsfoot trefoil (*Lotus corniculatus* L.), red clover (*Trifolium pretense* L.), and sweet clover (*Melilotus officinalis* L.). Taproot protein was positively associated with winter hardiness among cultivars and selections differing in fall dormancy (Cunningham et al., 2001). A major constituent of the taproot protein pool, vegetative storage proteins (VSPs), accumulate in fall and are preferentially depleted in spring when shoot growth resumes (Cunningham and Volenec, 1996). The extensive mobilization of VSPs and utilization of this N in new shoot growth were verified using ^15^N labeling (Avice et al., 1996; Barber et al., 1996). However, taproots of winter hardy and non-hardy alfalfa cultivars generally contained similar VSP concentrations (Cunningham and Volenec, 1996; Lu et al., 2018). This suggests that, while VSPs serve as an important source of N and are important for initial shoot regrowth in spring or after harvest, they may not control alfalfa winter hardiness *per se*.

Few studies have determined the impact of P and K nutrition on alfalfa taproot reserves, especially in the context of cold acclimation in fall and subsequent spring growth. In general, adequate P and K nutrition increases net photosynthesis (Cooper et al., 1967; Wolf et al., 1976; Dietz and Foyer, 1986; Hart and Greer, 1988; Tighe-Neira et al., 2018; Hu et al., 2019) and enhances carbohydrate assimilation and transport to storage organs (Conti and Geiger, 1982; Foyer and Spencer, 1986; Wang et al., 2018). In a greenhouse study, Li et al. (1997) observed low concentrations of starch and protein, and low VSP levels in taproots of K-deficient alfalfa plants, traits that were associated with poor survival of defoliated plants. In a related greenhouse experiment, Li et al. (1998) found that taproots of P-sufficient plants had slightly lower starch concentrations, but mobilized these starch reserves more extensively after defoliation and had more rapid shoot regrowth when compared to P-deficient plants. Taproot protein concentrations also declined rapidly in high-P plants when compared to those not supplied P. Using cluster analysis, Lissbrant et al. (2010) reported that taproots of K- and P-deficient plants sampled in the field in May had lower concentrations of protein and amino-N when compared to plants receiving adequate P and K. Small differences in taproot sugar and starch concentrations were observed in this study even though variation in P and K fertility resulted in large forage yield differences. Survival of alfalfa was poorest when K-deficient plants were fertilized with P. Most plants died between May and December, and this was associated with low concentrations of proteins and amino-N in taproots (Berg et al., 2018). Potassium deficiency results in low N_2_-fixation, decreases nodule number, and reduces photosynthesis and C transport to nodulated roots (Duke et al., 1980; Collins and Duke, 1981; Barta, 1982; Collins et al., 1986; Rao et al., 1990).

Our understanding of P and K concentrations in taproots of perennial legumes and how this relates to plant growth, persistence, and reserve accumulation and use is limited. This is despite the fact that taproots and crowns are the primary tissue remaining after harvest in summer and surviving overwinter. Taproot P concentrations increase gradually between early fall and late winter in alfalfa, birdsfoot trefoil, red clover, and sweet clover (Li et al., 1996). This change was accompanied by as much as a 3-fold increase in taproot phytate. Taproot P and phytate both declined substantially when shoot growth resumed in spring. By comparison, taproot K concentrations of these species generally declined in late fall (except red clover) and then slowly increased from December to May for all species. Unlike taproot P, there was no reduction in K concentrations when shoot growth resumed suggesting that K storage in taproots and remobilization to shoots do not occur in these species. Berg et al. (2018) compared taproot P and K concentrations to shoot P and K concentrations of clusters created by varied P and K fertilizer applications. Across a nearly 8-fold range in P concentrations, they found that taproot and shoot P concentrations were generally similar, with taproot levels being on average about 80% of shoot concentrations. By comparison, the 5-fold range in taproot K concentration was, on average, 28% those observed in shoots suggesting preferential partitioning of K to shoots over taproot tissues.

We hypothesize that the application of P and K fertilizer will enhance accumulation of specific taproot C and N reserve pools throughout the fall and that higher concentrations of these root reserves will improve winter hardiness and/or increase spring shoot growth of alfalfa. Our objectives were to: (1) determine seasonal patterns for taproot P and K concentrations during fall acclimation and during initial shoot growth in spring; (2) determine how P and K nutrition impacts accumulation of taproot C and N reserves during fall and their subsequent use when shoot growth resumes in spring; and (3) assess how addition of P and K fertilizer impacts plant survival and shoot growth in spring.

## Materials and Methods

### Field Design and Sampling

In April 1997, a 1.4-ha site at the Throckmorton Purdue-Agricultural Center located 15 km south of West Lafayette, IN, was seeded to Pioneer Brand “5454” alfalfa. This site was selected for study because soil tests indicated low concentrations of extractable P (9 to 15 mg kg^−1^) using the Bray P_1_ method (Bray and Kurtz, 1945; Sims, 2009) and low to moderate levels of exchangeable K (108 to 138 mg kg^−1^) according to Culman et al. (2020). Herbage was removed with a flail-type chopper and discarded four times in summer at approximately monthly intervals beginning in late May 1998 through late July 2001. In early September 2001, 10-m by 12.2-m field plots were established with four fertility treatments that represented the extreme P and K rates used in previous fertility research at this site (Berg et al., 2005, 2007, 2009, 2018). Four replicates of each treatment (0K-0P, 0K-75P, 400K-0P; and 400K-75P, all in kg P or K ha^−1^ yr^−1^) were arranged in a randomized complete-block design. The soil was a Lauramie silt loam soil (fine-loamy, mixed, active, mesic, and Mollic Hapludalf). Fertilizers were first applied in November 2001 and following the first (approximately May 25) and last (approximately September 10) herbage harvests of the 2002 and 2003 growing seasons, with half of the specified amount in each application. Chemical control of insects occurred when threshold limits were surpassed. Herbage yields were obtained with a flail-type chopper that harvested a 0.9-m-wide strip for approximately 10 m from the center of each plot. A random subsample of shoots was counted, dried at 70°C for 48 h, weighed, and dry mass per shoot calculated. The moisture concentration of this subsample was used to adjust field fresh weights of herbage yield to a dry matter basis. Shoots m^−2^ were estimated by dividing plot dry mass m^−2^ by the dry mass per shoot. Following herbage removal in May, taproots and crowns in a 0.5 m^2^ area were excavated, counted, and plants m^−2^ calculated.

### Taproot Sampling

To study C and N reserve patterns during fall acclimation, taproots were sampled following the final annual forage harvest on September 7, 2002 and 2003 (Day 0) and at intervals thereafter (Days 7, 14, 21, 28, 42, 56, 70, and 84). To understand the use of taproot C and N in spring growth initiation, additional taproots were sampled beginning March 27 of 2003 and 2004 (Day 0) and continued weekly (Days 7, 14, 21, 28, 35, 42, and 49) until the first herbage harvest (approximately May 25). Approximately 20 taproots (measuring ~0.2-m in length) were excavated from a randomly selected area within each plot and washed free of soil in cold water. Each taproot was divided into two segments; the uppermost 5 cm of taproot immediately below the crown was cut and separated for RNA extraction, while the remainder of the taproot (5 to 20 cm) was retained for analysis of the N and C pools. Previous reports (Escalada and Smith, 1972) indicated that sugar and starch concentrations in 5-cm segments of alfalfa taproots responded similarly down to 30 cm. The top 5 cm of taproot was diced into small pieces and immersed immediately in liquid N_2_. These root samples were stored at −80°C until total RNA was extracted. The remainder of the taproot was cut into 2-cm-long segments, frozen on solid CO_2_, and lyophilized. Taproot tissues were ground to pass a 1-mm screen and stored at −20°C.

### P and K Analyses

Taproot tissues (~350 mg) were digested in 5 ml of 15.8 mol L^−1^ HNO_3_ in 50 ml digestion tubes using an adjusted micro-Kjeldahl digestion method (Nelson and Sommers, 1973). Samples were mixed, placed on a preheated digestion block at 175°C until the solution boiled, and then immediately removed and allowed to cool. After cooling, 4 ml of 30% (v/v) H_2_O_2_ was added to each tube, and the samples were returned to the heating block at 175°C. When boiling resumed, tubes were removed and allowed to cool. The process of adding 4 ml of 30% (v/v) H_2_O_2_ was repeated until tissues were completely digested. Volumes were adjusted to 50 ml with deionized, reverse-osmosis water and samples mixed. Mineral analyses were conducted using an inductively coupled plasma spectrophotometer.

### Sugar and Starch Analyses

Sugars were extracted from 30 mg of freeze-dried taproot tissue with 1 ml of 800 ml L^−1^ ethanol in 1.5-ml microfuge tubes. Tubes were shaken for 10 min at 25°C, microfuged at 14,000 *g_n_* for 5 min at 4°C, and the supernatant retained. The ethanol extraction was repeated twice, and the combined supernatants diluted to a final volume of 10 ml with ethanol (800 ml L^−1^). Sugar concentrations in the ethanol extracts were determined with anthrone (Sigma Chemical Co., St. Louis, MO, United States) using glucose (Mallinckrodt Chemical Works, St. Louis, MO, United States) as a standard (Van Handel, 1968). The ethanol-extracted residue was oven-dried at 55°C. Water (500 μl) was added to each tube, and the tubes were heated in a boiling water bath for 10 min to gelatinize starch. The pH of the solution was adjusted to 5.1 by adding 400 μl 0.2 N Na acetate buffer (Sigma Chemical Co., St. Louis, MO, United States). Starch was digested by adding 0.2 U of amyloglucosidase (Sigma Chemical Co., St. Louis MO; product A3514 from *Aspergillus niger*) and 40 U of α-amylase (Sigma Chemical Co., St. Louis MO; product A0273 from *Aspergillus oryzae*) in 100 μl of 0.2 N Na acetate buffer (pH 5.1). Tubes were incubated at 55°C for 24 h with occasional shaking. Tubes were centrifuged as before, and glucose in the supernatant was determined using glucose oxidase (Glucose Trinder, Sigma Chemical Co., St. Louis MO; Product 315-100). Starch concentration was estimated as 0.9 × glucose concentration.

### Protein, Amino Acid, and RNA Analyses

Protein analysis was conducted at 4°C unless otherwise stated. Soluble proteins were extracted by suspending 30 mg of freeze-dried taproot tissue in 1 ml of 100 mM sodium phosphate (J.T. Baker, Phillipsburg, NJ, United States) buffer (pH 6.8) containing 1 mM phenylmethylsulfonylfluoride (Aldrich Chemical Co., St. Louis, MO, United States) and 10 mM 2-mercaptoethanol (Sigma Chemical Co., St. Louis, MO, United States). Tissue suspensions were vortexed four times for 30 s at 5-min intervals and then centrifuged at 14,000 *g_n_* for 10 min. The supernatants were retained. Soluble protein in the supernatants was estimated using protein dye-binding (Bradford, 1976). Amino acids in the supernatant were analyzed with ninhydrin (Sigma-Aldrich Chemical Co., St. Louis, MO, United States; Rosen, 1957) using glycine (Sigma Chemical Co., St. Louis, MO, United States) as a standard. RNA was extracted as described by Ougham and Davis (1990) by grinding the uppermost 5 cm of the taproot in liquid nitrogen using a motar and pestle. The finely ground tissues were suspended in 4 ml water-saturated phenol (Sigma Chemical Co., St. Louis, MO, United States) at 65°C followed by 4 ml of extraction buffer [0.2 M sodium acetate, pH 5.2, 10 mM disodium EDTA (Mallinckrodt Specialty Chemicals, Paris, KY, United States), 1% (w/v) sodium dodecylsulfate (Sigma Chemical Co., St. Louis, MO, United States)] at 65°C. Following precipitation with LiCl (Sigma Chemical Co., St. Louis, MO, United States), the RNA pellets were washed with ethanol, dried, and carefully re-suspended in a known volume of sterile water. RNA concentrations were determined based on absorbance at 260 nm and total sample volume.

### Statistical Analysis

Statistical analysis of the randomized complete-block design included harvest as subplots in a repeated measures analysis using the Proc Mixed routine in SAS (version 9.4, SAS Institute Inc., 2015). The Akaike Information Criterion was used to select the proper covariance structure (autoregressive, compound symmetry, and unstructured) for each analyte (Elliott and Woodward, 2016). Years were considered as random, and fall acclimation vs. spring growth datasets were analyzed separately. Variation was partitioned into fertility treatment and sampling date main effects, and the fertility treatment by sampling date interaction. Differences among least squares means at *p* < 0.05 were determined using the LSMEANS/PDIFF procedure in SAS. Herbage yield and yield components sampled in May of 2002 to 2004 were analyzed by year using ANOVA. Where the F-test was significant (*p* ≤ 0.05), the least significant difference (LSD) was used to compare means.

## Results

### Weather

**Figure 1:**
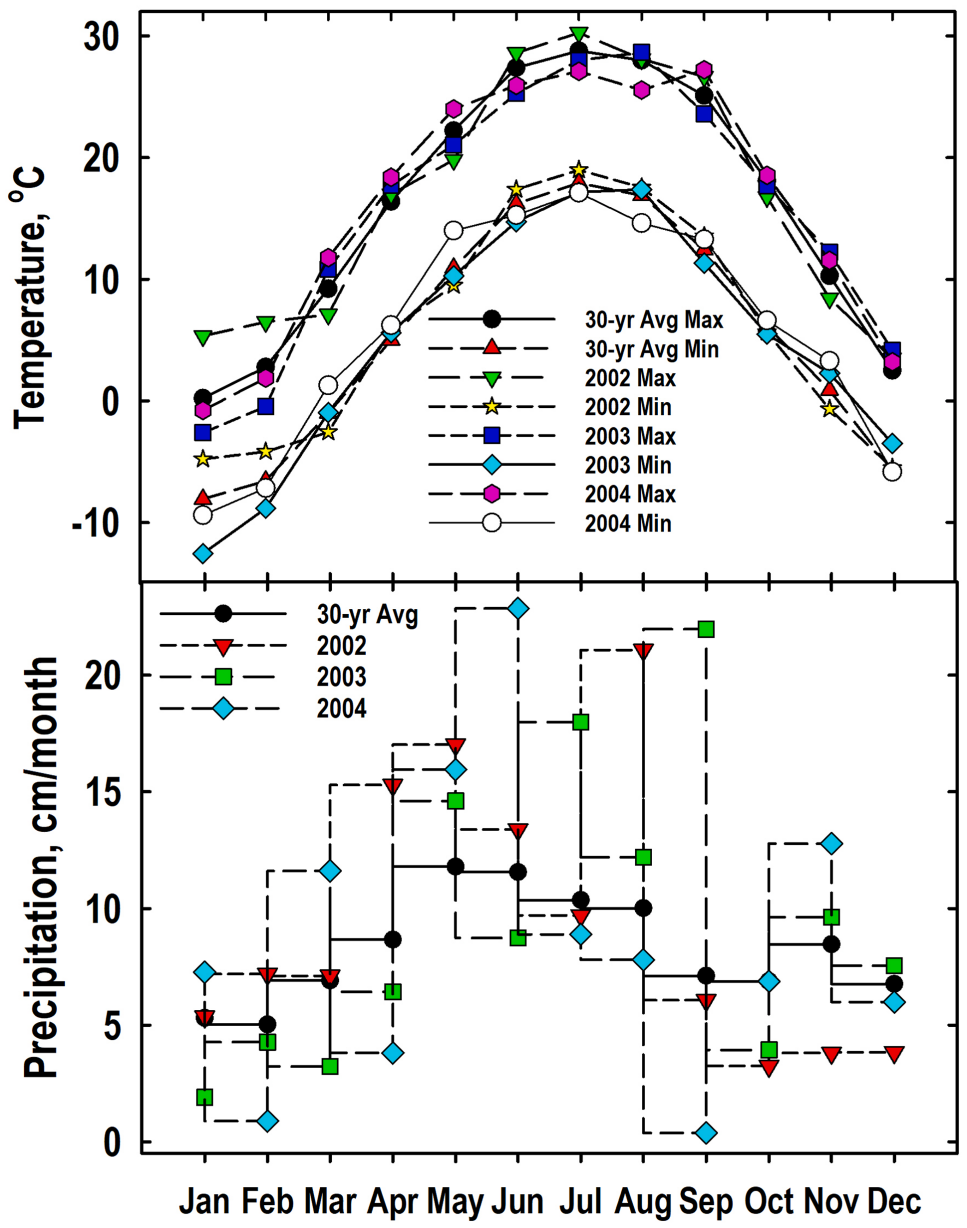
Monthly values for mean air temperatures (maximum and minimum) and precipitation from 2002 to 2004 at the Throckmorton Purdue Agricultural Center. For comparison, the 30-year average temperatures and precipitation values also are provided.

Patterns for both maximum and minimum air temperatures generally followed the 30-yr trend (Figure 1). Exceptions when the plant sampling occurred between September 2002 and May 2004 include a cooler-than-normal temperatures in January and February 2003. As expected, precipitation was more variable than temperature. Months that were notably wetter than the long-term average include April, May, and August of 2002; July and September of 2003; and March and June of 2003. Months notable drier than the 30-yr average included November and December of 2002, January and March of 2003, and February, April, and September of 2004.

### Taproot Mineral Concentrations

**Figure 2:**
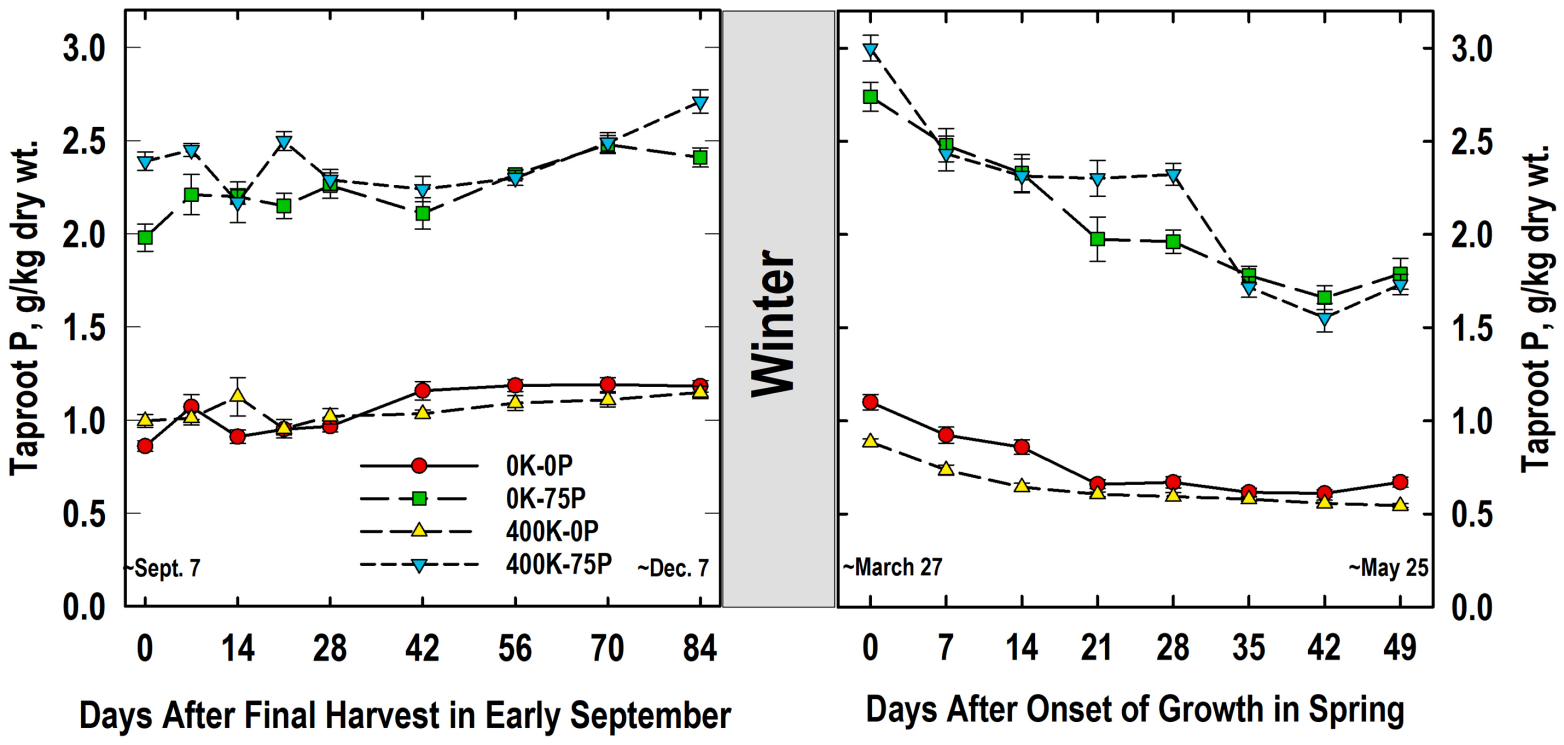
Concentration of phosphorus (P) in taproots of alfalfa during fall acclimation and following resumption of shoot growth in spring. Plants were fertilized with P (0 or 75 kg P ha^−1^ yr^−1^) and K (0 or 400 kg K ha^−1^ yr^−1^) and harvested four times annually during summer. Following the final harvest on September 7, taproots were sampled weekly for a month, then on alternate weeks until December 7. Taproots were sampled weekly in spring until shoot harvest on May 25. Data were averaged across two fall–winter–spring seasons (2002–2003 and 2003–2004). Error bars represent one standard error (SE) of the mean (*N* = 8).

As expected, plants fertilized with P (400K-75P, 0K-75P) always had significantly higher taproot P concentrations than those not fertilized with P, and this response was not influenced by K fertilization (Figure 2). The P concentrations of plants fertilized with 400K-75P were 2.4 g P kg^−1^ dry wt. at defoliation in early September (Day 0) and remained relatively constant until increasing between Days 56 and 84. This same trend was observed for the 0K-75P treatment with concentrations observed from Days 56 to 84 exceeding those observed on Day 0; however, P levels were initially lower in the 0K-75P treatment. The P concentrations of plants not fertilized with P were low and initially similar (~0.9 g P kg^−1^ dry wt.) and remained unchanged during fall for the 400K-0P treatment, while increasing over Day 0 values on Day 42 and thereafter for the 0K-0P treatment.

Little change in P concentrations of treatments occurred overwinter, and the relative ranking of the treatments in March was unchanged (Figure 2). The P concentrations of plants not fertilized with P (0K-0P, 400K-0P) were similar at all samplings, while P concentrations of P-fertilized plants were similar irrespective of K fertilizer application except on Day 28 when K fertilization increased taproot P. On Day 0, plants fertilized with P contained approximately 3 g P kg^−1^ dry wt. and these declined until Day 14 (400K-75P) or 21 (0K-75P), followed by a second decline for both treatments after Day 28. Initial P concentrations of P-unfertilized treatments averaged 1 g P kg^−1^ dry wt. on Day 0 when growth resumed and, like the P-fertilized plants, declined significantly by Days 21 (0K-0P) and 28 (400K-0P) remaining relatively unchanged thereafter.

**Figure 3:**
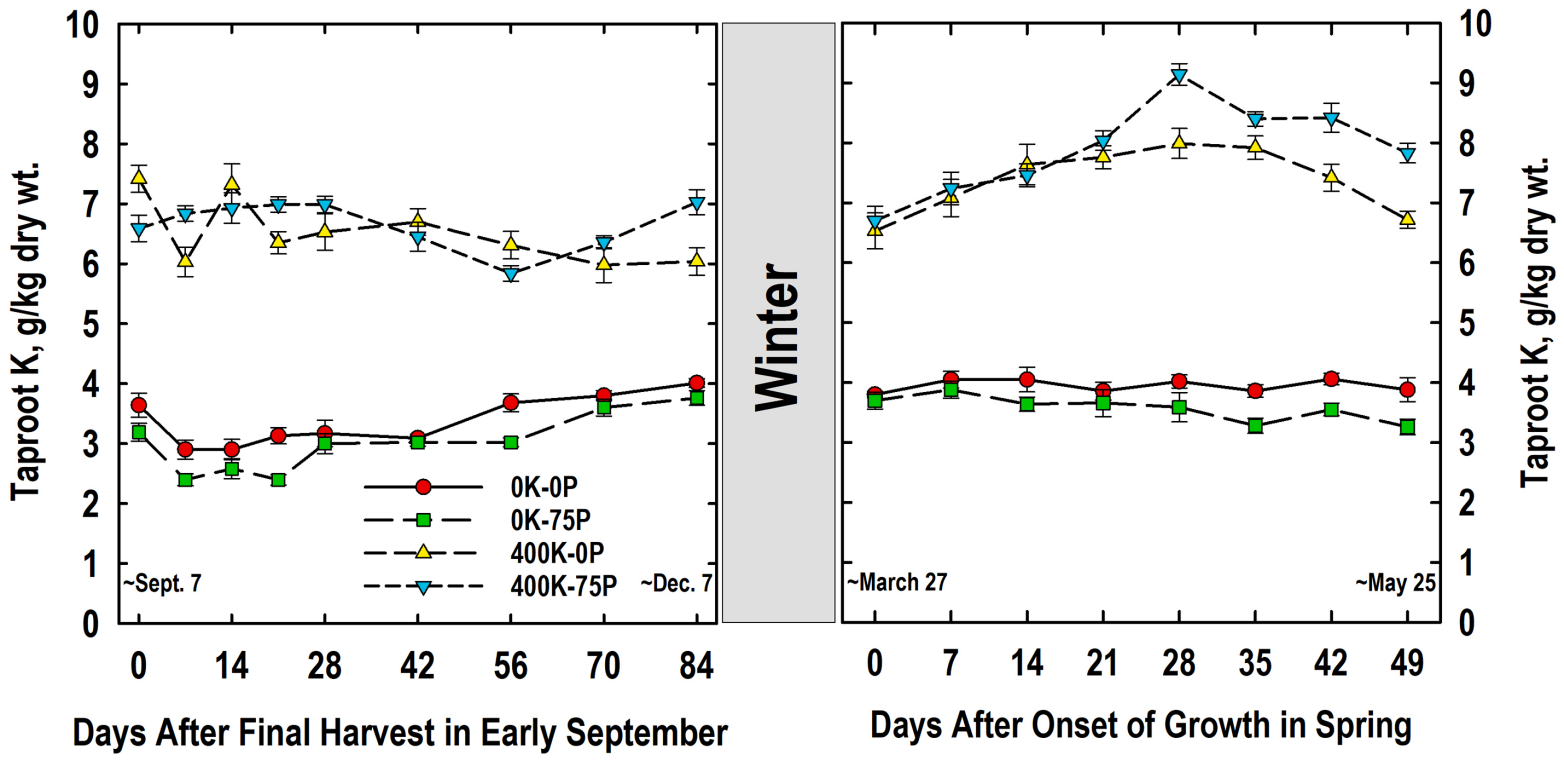
Concentration of potassium (K) in taproots of alfalfa during fall acclimation and following resumption of shoot growth in spring. Plants were fertilized with P (0 or 75 kg P ha^−1^ yr^−1^) and K (0 or 400 kg K ha^−1^ yr^−1^) and harvested four times annually during summer. Following the final harvest on September 7, taproots were sampled weekly for a month, then on alternate weeks until December 7. Taproots were sampled weekly in spring until shoot harvest on May 25. Data were averaged across two fall–winter–spring seasons (2002–2003 and 2003–2004). Error bars represent one standard error (SE) of the mean (*N* = 8).

Taproot K concentrations were approximately two-fold higher in K-fertilized plants when compared to plants not fertilized with K, a difference that was not influenced by P-fertilizer application (Figure 3). Tissue K concentrations varied little during fall, but were significantly lower on Day 56 for the 400K-75P than values on Days 14 to 28, while the K concentration on Day 0 of the 400K-0P treatment was higher than those observed on Days 7, 21, and 56 to 84. For plants not fertilized with K, K concentrations were statistically similar on Days 0 and 7, then increased gradually after Day 21, and were significantly higher than Day 7 values for both treatments by Day 84.

The following March, K concentrations were similar to those observed in December of the previous year, with higher concentrations in taproots of plants fertilized with K (Figure 3). The K levels in K-fertilized plants increased significantly between Day 0 and 14, and peaked on Day 28 before declining significantly by Day 49. Concentrations for the 400K-75P treatment were greater than the 400K-0P treatment on Days 28, 42, and 49. By comparison, K concentrations in plants not fertilized with K were always low, not altered by P-fertilization, and concentrations did not change significantly between Days 0 and 49.

### Taproot Carbon Pools

**Figure 4:**
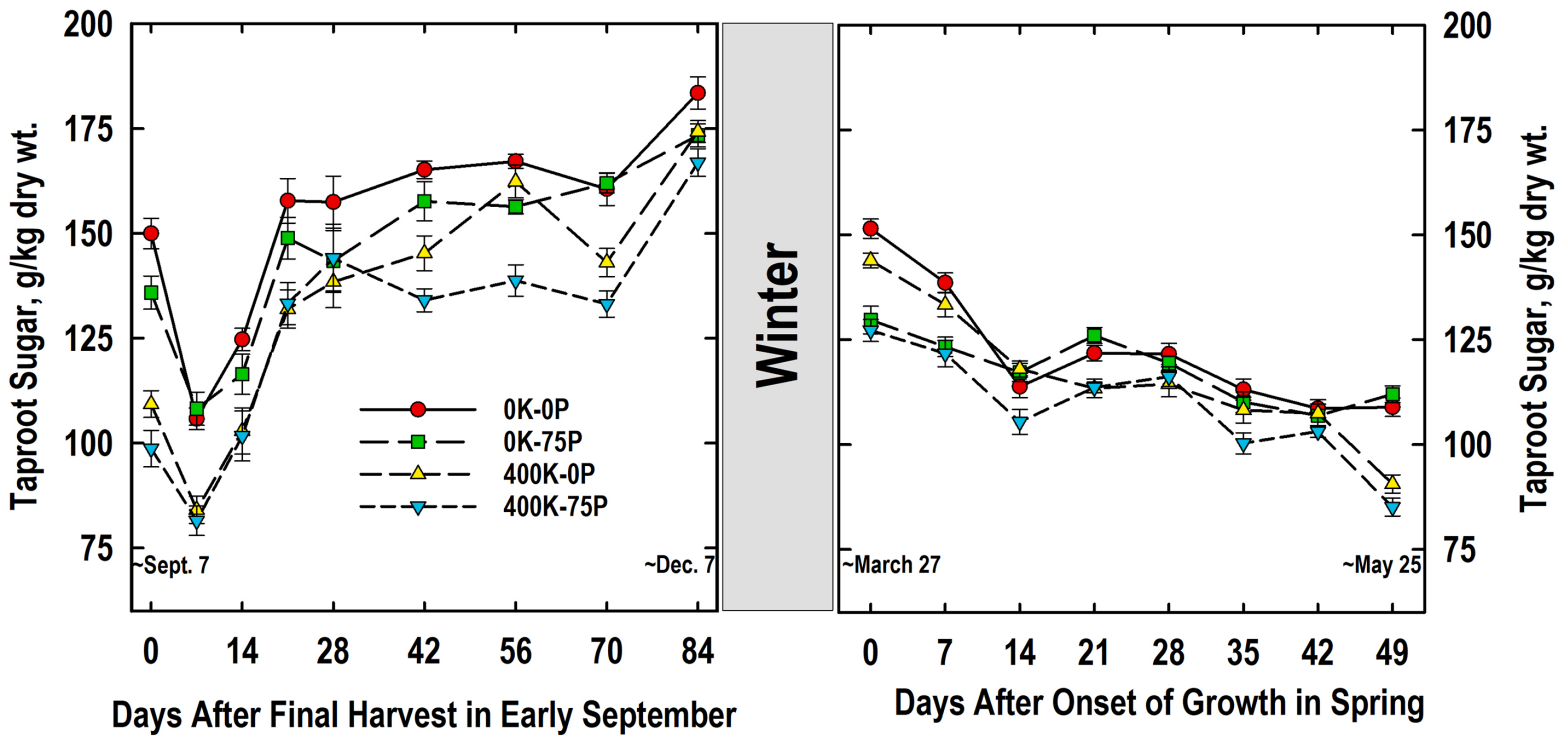
Concentration of ethanol-soluble sugar in taproots of alfalfa during fall acclimation and following resumption of shoot growth in spring. Plants were fertilized with P (0 or 75 kg P ha^−1^ yr^−1^) and K (0 or 400 kg K ha^−1^ yr^−1^) and harvested four times annually during summer. Following the final harvest on September 7, taproots were sampled weekly for a month, then on alternate weeks until December 7. Taproots were sampled weekly in spring until shoot harvest on May 25. Data were averaged across two fall–winter–spring seasons (2002–2003 and 2003–2004). Error bars represent one standard error (SE) of the mean (*N* = 8).

Taproot sugar concentrations of plants in the 0K-0P and 0K-75P treatments were initially higher than those of plants fertilized with K and exhibited a significant reduction after defoliation on Day 0 (Figure 4). Sugar concentrations were reduced in taproots of K-fertilized plants, but this reduction was only statistically significant for the 400K-0P treatment. Sugar accumulated to significantly higher concentrations by Day 21 for all treatments, during which time concentrations for the 0K-0P treatment always exceed that of both K-fertilized treatments. Sugar concentrations for all treatments varied little between Days 21 and 70, and except for Day 28, differences between the 0K-0P and 400K-75P treatments persisted. A significant and abrupt increase in sugar occurred in taproots between Days 70 and 84 for all except the 0K-75P treatment, a response common as plants harden for winter.

Taproot sugar concentrations declined overwinter for all treatments, with concentrations for the 400K-75P and 0K-75P treatments being similar to each other, and lower than the 0K-0P and 400K-0P treatments on Days 0 and 7 (Figure 4). As shoot growth resumed, significant reductions in taproot sugar occurred for all treatments except 0K-75P by Day 14. Sugar concentration of all treatments was similar between Days 14 and 42, but declined on Day 49 for both K-fertilizer treatments re-establishing the relative treatment ranking observed when plants were first sampled the previous September.

**Figure 5:**
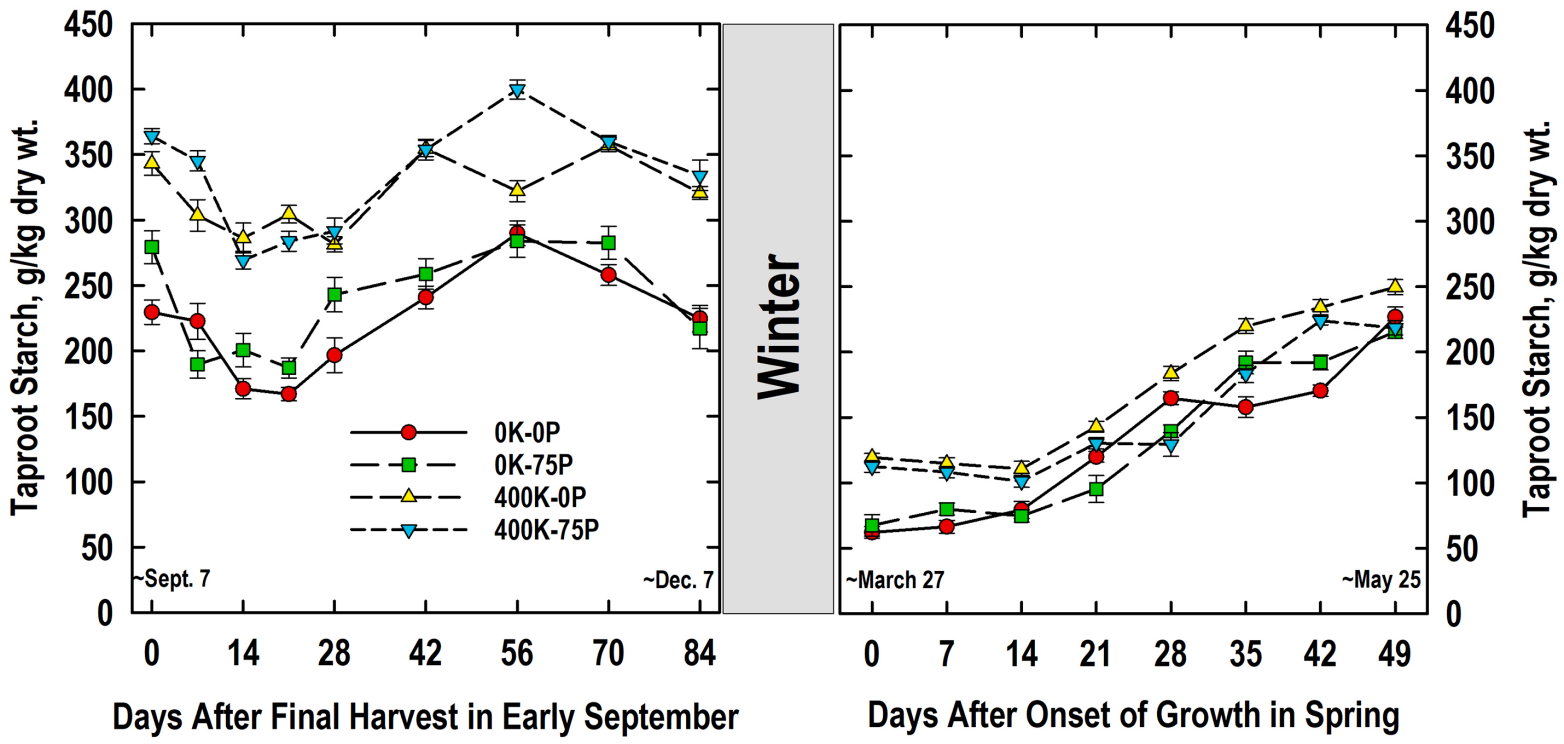
Concentration of starch in taproots of alfalfa during fall acclimation and following resumption of shoot growth in spring. Plants were fertilized with P (0 or 75 kg P ha^−1^ yr^−1^) and K (0 or 400 kg K ha^−1^ yr^−1^) and harvested four times annually during summer. Following the final harvest on September 7, taproots were sampled weekly for a month, then on alternate weeks until December 7. Taproots were sampled weekly in spring until shoot harvest on May 25. Data were averaged across two fall–winter–spring seasons (2002–2003 and 2003–2004). Error bars represent one standard error (SE) of the mean (*N* = 8).

In contrast to taproot sugar accumulation, starch concentrations of K-fertilized plants were generally higher than those observed in plants not fertilized with K (Figure 5). Defoliation on Day 0 resulted in lower starch concentrations in all treatments by Day 14. Starch concentrations remained unchanged for all treatments between Days 14 and 21, and then increased significantly by Day 42. Between Days 56 and 84, significant reductions in starch concentrations were observed in all treatments except the 400K-0P, an trend coinciding with the increases in taproot sugar accumulation that often accompanies with winter hardening (Figure 4).

Large reductions in taproot starch occurred overwinter, and the treatment ranking observed when growth resumed in March corresponded well to that observed the previous December (Figure 5). Irrespective of P fertilization, K-fertilized plants had higher starch on Days 0 and 7 than plants not fertilized with K. Thereafter, starch concentrations of the 400K-0P treatment were higher than that of the 0K-0P (Days 14, 35, and 42) and 0K-75P (Days 14 to 21, 42, and 49) treatments. Starch concentrations were more variable in the 400K-75P treatment where concentrations were greater than the 0K-75P treatment on Days 21 and 42 and the 0K-0P treatment on Day 42, but lower than this treatment on Day 28. With the exception of Day 35, starch concentrations were similar at all samplings of the 0K-0P and 0K-75P treatments.

### Taproot Nitrogen Pools

**Figure 6:**
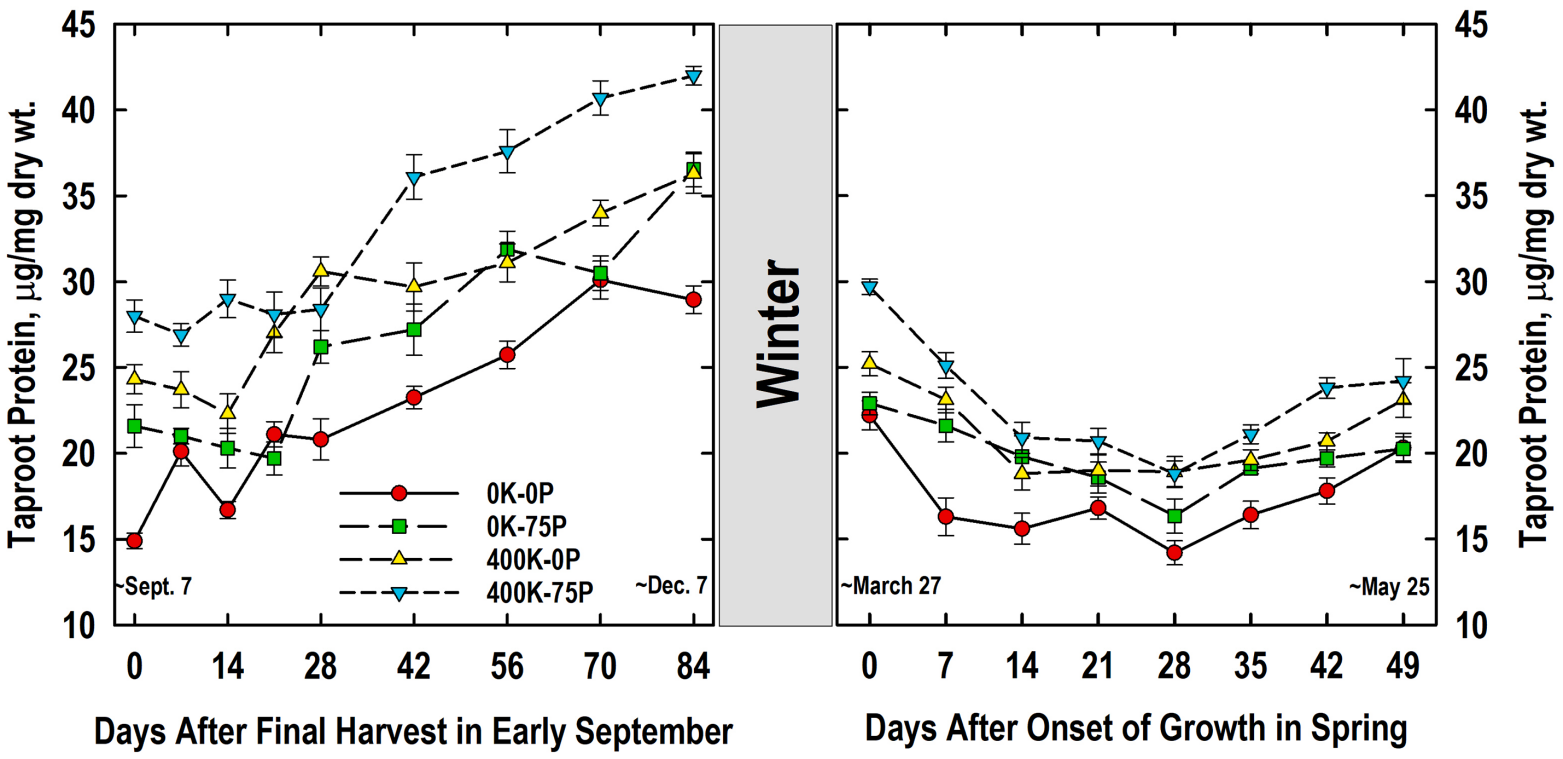
Concentration of buffer-soluble protein in taproots of alfalfa during fall acclimation and following resumption of shoot growth in spring. Plants were fertilized with P (0 or 75 kg P ha^−1^ yr^−1^) and K (0 or 400 kg K ha^−1^ yr^−1^) and harvested four times annually during summer. Following the final harvest on September 7, taproots were sampled weekly for a month, then on alternate weeks until December 7. Taproots were sampled weekly in spring until shoot harvest on May 25. Data were averaged across two fall–winter–spring seasons (2002–2003 and 2003–2004). Error bars represent one standard error (SE) of the mean (*N* = 8).

Taproot protein concentrations were higher in the 400K-75P treatment than the 0K-0P and 0K-75P treatments from Days 0 to 21 and Days 42 to 84 (Figure 6). The 400K-0P treatment also had lower taproot protein levels than the 400K-75P treatment on Days 14, and 42 to 84. Compared to initial levels on Day 0, significantly higher protein concentrations were observed on Days 21 (0K-0P), 28 (400K-0P), and 42 (0K-75P, 400K-75P), increases that continued until Days 70 (0K-0P) and 84. Final concentrations on Day 84 were highest in the 400K-75P treatment, lowest in the 0K-0P treatment, and intermediate in the others.

Taproot protein concentration declined overwinter (Figure 6). The 400K-75P treatment maintained its advantage in protein concentration over the 0K-0P treatment throughout growth in spring with the exception of Day 49. Similarly, the 400K-0P treatment had higher taproot protein concentrations than the 0K-0P treatment on Days 7, 28, 35, and 42 (*p* = 0.08). The 0K-75P also had higher taproot protein than the 0K-0P treatment on Day 7, but had lower taproot protein than the 400K-75P on Day 42. Protein concentrations declined as shoot growth resumed and continued until Day 7 (0K-0P), 14 (400K-0P, 400K-75P), or 28 (0K-75P). For most treatments the lowest protein concentrations were observed in taproots on Day 28, after which significant increases occurred by Day 35 (0K-75P) or 42 (0K-0P, 400K-75P), the exception being 400K-0P where no significant increase in taproot protein concentration occurred after the lowest level observed on Day 14.

**Figure 7:**
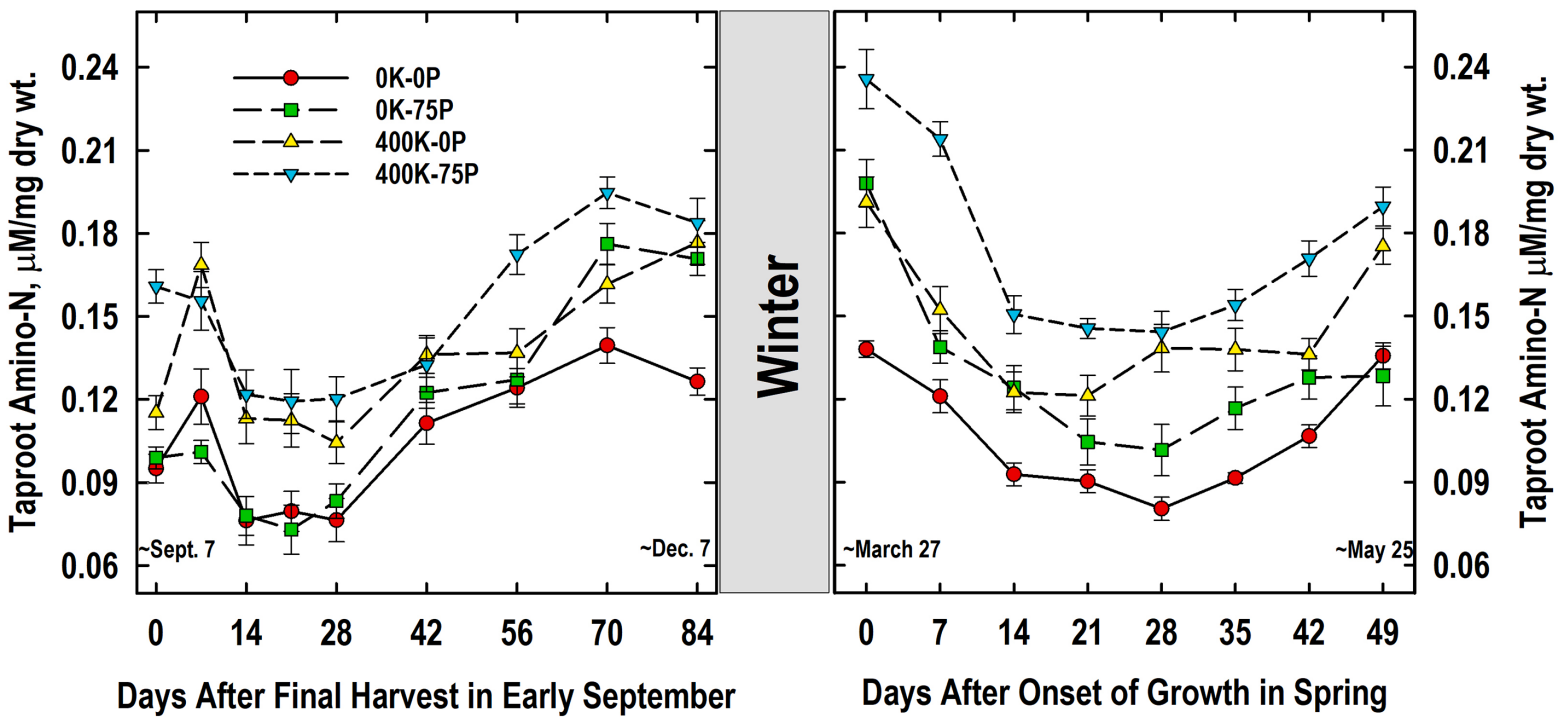
Concentration of amino acids in taproots of alfalfa during fall acclimation and following resumption of shoot growth in spring. Plants were fertilized with P (0 or 75 kg P ha^−1^ yr^−1^) and K (0 or 400 kg K ha^−1^ yr^−1^) and harvested four times annually during summer. Following the final harvest on September 7, taproots were sampled weekly for a month, then on alternate weeks until December 7. Taproots were sampled weekly in spring until shoot harvest on May 25. Data were averaged across two fall–winter–spring seasons (2002–2003 and 2003–2004). Error bars represent one standard error (SE) of the mean (*N* = 8).

Taproot amino-N concentrations at defoliation in September were also higher in the 400K-75P treatment when compared to the other fertilizer treatments (Figure 7). Amino-N concentrations of the 400K-75P were unchanged on Day 7 and then declined on Days 14 to 28 before increasing significantly by Day 56 through 84. Taproots of plants of the other treatments exhibited this same pattern of low concentrations between Days 14 and 28 followed by significantly higher concentrations from Days 56 to 84 (Days 70 to 84 for the 400K-0P treatment). Taproot amino-N concentrations of the 0K-0P and 0K-75P treatments were similar at all samplings except on Days 70 (*p* = 0.09) and 84 when P fertilization alone increased amino-N levels. Taproot amino-N concentrations were similar in the 400K-0P and 400K-75P treatments with the exception of Days 0 and 56 (*p* = 0.10).

Taproot amino-N concentrations did not change markedly overwinter, and the relative ranking of concentrations in March was similar to the previous December (Figure 7). Amino-N in late March declined significantly by Day 7 in the 0K-75P treatment and by Day 14 in all others. Low amino-N concentrations were maintained until Day 28 for the 0K-0P, 0K-75P, and 400K-75P treatments before increasing significantly on Day 42 and thereafter. The low amino-N levels observed on Day 14 persisted until Day 42 in the 400K-0P treatment, increasing significantly on Day 49. Amino-N concentrations of the 400K-75P treatment were greater than all other treatments on Days 0 and 7. At subsequent samplings, the 0K-0P and 0K-75P treatments had lower amino-N concentrations than the 400K-75P treatment with the exception of Day 14 when the 400K-75P and 0K-75P treatments were similar. With the exception of Day 0, amino-N concentrations of the 0K-0P and 0K-75P treatments were similar throughout spring samplings. Although numerically lower at all samplings, amino-N concentrations of the 400K-0P treatment were statistically similar to those of the 400K-75P treatment from Day 14 onward except Day 42 (*p* = 0.08).

### Taproot RNA Pools

**Figure 8:**
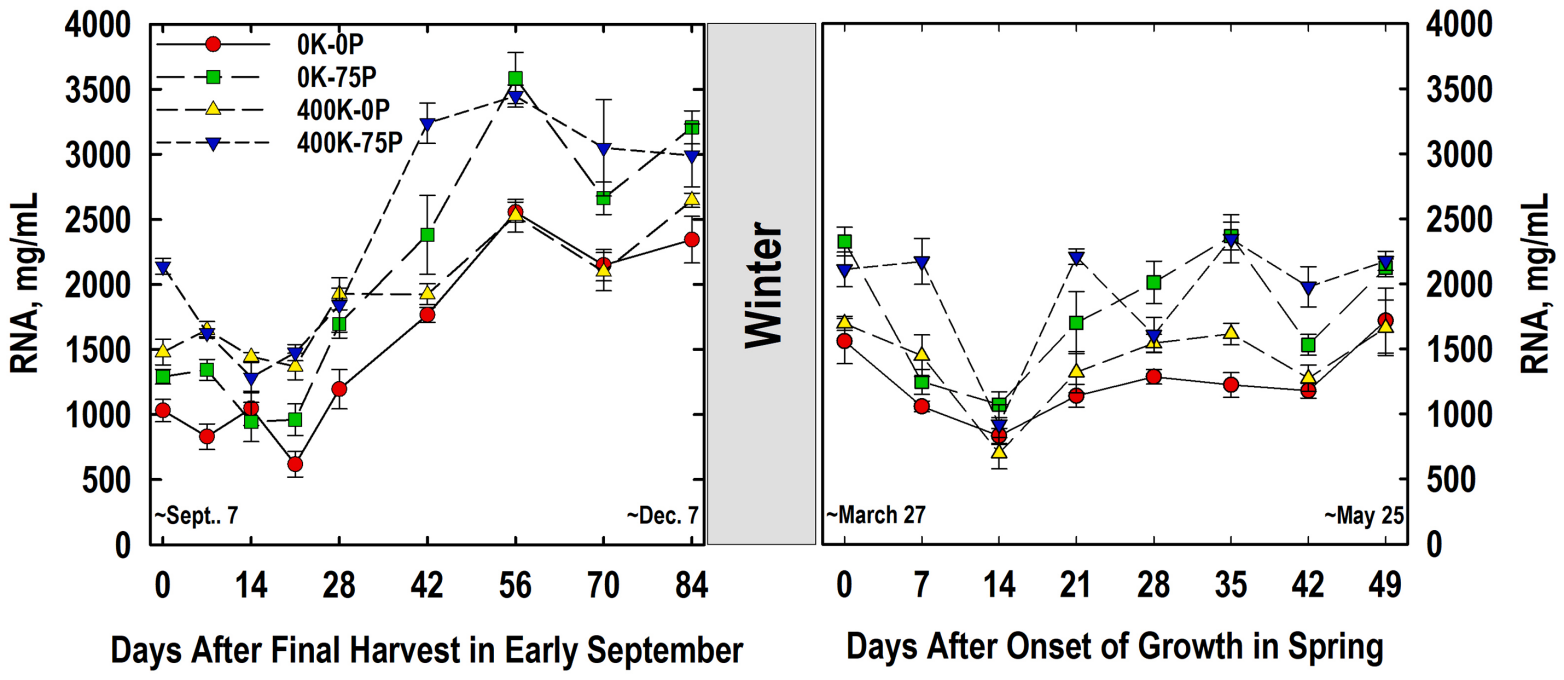
Concentration of RNA in taproots of alfalfa during fall acclimation and following resumption of shoot growth in spring. Plants were fertilized with P (0 or 75 kg P ha^−1^ yr^−1^) and K (0 or 400 kg K ha^−1^ yr^−1^) and harvested four times annually during summer. Following the final harvest on September 7, taproots were sampled weekly for a month, then on alternate weeks until December 7. Taproots were sampled weekly in spring until shoot harvest on May 25. Data were obtained from the 2003–2004 fall–winter–spring season only. Error bars represent one standard error (SE) of the mean (*N* = 4).

Results from RNA analysis in Year 1 suggested that fertilizer treatment and sampling time affected taproot RNA concentrations. Unfortunately, slight differences in extraction/re-suspension volumes precluded us from accurately quantifying RNA levels in Year 1. RNA concentrations were higher in the 400K-75P treatment when taproots were sampled at defoliation in early September of Year 2 (Figure 8). These RNA concentrations in taproots of plants in the 400K-75P treatment declined significantly by Day 14, followed by a large increase on Day 42 and thereafter. The other fertilizer treatments also exhibited significant increases in RNA concentrations after Day 21, but this was not preceded by a significant decline after defoliation on Day 0. The RNA concentrations in taproots of the 0K-0P were initially lower than those of the 400K-0P treatment on Days 7, 21, and 28, but RNA levels of these treatments not fertilized with P were similar thereafter, and as a group, lower than RNA concentrations observed in taproots of P-fertilized plants irrespective of K fertilizer application.

Taproot RNA levels in March were slightly lower than concentrations observed the preceding December, but the relative ranking of treatments was consistent (Figure 8). On Day 0, taproots of plants not fertilized with P had lower RNA concentrations than plants fertilized with 0K-75P. The RNA concentrations declined significantly by Day 7 (0K-75P) or 14 (others), before increasing by Day 21 for all treatments except plants in the 0K-0P regime. The RNA concentrations observed for the 0K-0P treatment on Day 49 were higher than those on Day 14. Taproot RNA concentrations were similar at all spring samplings for the 0K-0P and 400K-0P treatments. When compared to P-fertilized plants, RNA concentrations were significantly lower in the 0K-0P plants on Days 7, 21, 35, and 42 (vs. 400K-75P) and Days 0, 28, and 35 (vs. 0K-75P). On Day 49, RNA concentrations were statistically similar for all treatments, although numerically higher for P-fertilized plants.

### Plant Growth and Development

Fertilizer treatments applied in November 2001 increased forage yield in May 2002, the spring forage harvest prior to the onset of taproot sampling in September 2002 (Table 1). Yield of the 400K-75P and 0K-75P fertilizer treatments was similar and higher than that of the 0K-0P and 400K-0P treatments. In May of 2003, forage yield of the 0K-0P and 400K-0P plots was lower than the 0K-75P treatment that was, in turn, lower than plots fertilized with 400K-75P. In May 2004, forage yield of the 0K-0P and 400K-0P was again similar, the latter treatment had yields similar to the 0K-75P treatment, and all of these were lower than the 400K-75P treatment.

**Table 1:** Yield and yield components of alfalfa sampled in May of 2002, 2003, and 2004 for plants fertilized with four contrasting amounts of potassium (K) and phosphorus (P) fertilizer beginning September 2001.

Fertility treatment					
Characteristic	0K-0P	0K-75P	400K-0P	400K-75P	LSD
Yield, kg ha^−1^					
May 2002	2,031±222	3,097±298	2,438±103	3,407±116	455
May 2003	2,208±223	3,485±145	2,643±200	4,649±24	544
May 2004	1,648±238	3,159±306	2,402±171	5,676±235	779
Mass shoot^−1^, g					
May 2002	0.59±0.03	0.68±0.03	0.74±0.06	0.87±0.07	0.16
May 2003	0.60±0.07	0.93±0.06	0.90±0.07	1.39±0.10	0.22
May 2004	0.62±0.09	0.92±0.07	0.73±0.11	1.57±0.15	0.34
Shoots m^−2^					
May 2002	344±39	455±38	334±18	398±22	73^‡^
May 2003	373±26	379±14	304±44	339±24	ns^†^
May 2004	277±44	352±53	344±35	370±35	ns
Plants m^−2^					
September 2001	71±11	81±10	92±10	82±13	ns
May 2002	74±8	57±10	81±6	69±12	ns
May 2003	28±5	41±4	57±2	48±6	14
May 2004	41±14	31±7	44±8	45±5	ns

*Treatments included unfertilized control plots (0K-0P), high P only (0K-75P), high K only (400K-0P), and high K and P (400K-75P) where the numerals indicate the annual rate in kg ha^−1^. One-half of the shown fertilizer rate was applied in May after the first forage harvest and the remaining half after the last forage harvest in September of each year. Means (N = 4) and standard errors are provided. Unless otherwise indicated the least significant difference (LSD) is provided where F-tests were significant (p < 0.05)*.

† *not significant at p < 0.05.*

‡ *LSD at p ≤ 0.10.*

Differences in mass shoot^−1^ generally mirrored fertilizer treatment-induced trends in herbage yield (Table 1). In May 2002, mass shoot^−1^ of the 0K-0P and 0K-75P treatments was similar and lower than that of the 400K-75P treatment. In May 2003, mass shoot^−1^ was lowest in the 0K-0P treatment, highest in the 400K-75P treatment, with the other treatments intermediate. In May 2004, the 400K-75P treatment had higher mass shoot^−1^ than the other fertilizer treatments.

Shoots m^−2^ differed in May 2002 with fewer shoots present in plots fertilized with 0K-0P and 400K-0P when compared to 0K-75P (Table 1). Thereafter, shoot density was not influenced by fertilizer treatment and averaged 342 shoots m^−2^.

Fertility treatments had little effect on plant persistence. Plots had similar plant populations when fertilizer treatments were imposed in September 2001 averaging 82 plants m^−2^ (Table 1). Significant differences in plant populations were observed in May 2003 when plots of the 0K-0P had lower populations than the 400K-0P and 400K-75P plots, and the 0K-75P plots had few plants m^−2^ than the 400K-0P plots. However, these treatment-related differences disappeared by the termination of the study in May 2004 when plant populations had declined by about 50% (averaged 40 plants m^−2^).

## Discussion

Nearly a century of research has supported the premise that taproot carbohydrate and protein reserves provide C and N for growth and respiration of alfalfa during periods when photosynthesis and N_2_-fixation are reduced by defoliation in summer or other environmental stresses (Graber et al., 1927; Jung and Smith, 1961a; Avice et al., 1996; Volenec et al., 1996; Berg et al., 2018; Mitchell et al., 2020). Numerous studies have also shown that plant nutrition, and in particular P and K fertility, plays a key role in alfalfa growth and stress tolerance (Brown, 1928; Roberts and Olson, 1944; Jung and Smith, 1959; Cooper et al., 1967; Wolf et al., 1976; Kitchen et al., 1990; Volenec et al., 2021). In this study, herbage yield was greatest when both P and K were supplied (Table 1) indicating that initial levels of P and K in soil were insufficient to meet plant growth needs. In addition, mass/shoot was the yield component responsible for the higher forage yields. This is consistent with previous long-term studies focused on the impact of P and K nutrition on alfalfa yield at this site (Berg et al., 2005, 2007, 2009, 2018; Lissbrant et al., 2010). Lloveras et al. (2012) also reported that shoot weight was the yield component associated with K fertilizer-induced changes in alfalfa yield. This also agrees with a recent report by Jungers et al. (2019) who reported significant increases in forage yield at the Beck MN site that were largely independent of stem density; by default, these yield increases would have resulted from greater mass/shoot. The minor impact of P and K treatments on plant persistence (plants/m^2^) in this study that spanned 2 years is not surprising. Related research at this site revealed that 4 or more years of differential P and K fertilizer application was necessary before significant differences in plant persistence were observed, with greatest reductions in plant survival in plots fertilized with P but not K (Berg et al., 2018; Volenec et al., 2021). A trend for reduced plant populations for the 0K-75P treatment, when compared to K-fertilized plants, was evident at May 2002 to 2004 samplings, but differences were not statistically significant (Table 1).

As expected, application of P and K fertilizers increased concentrations of these nutrients in taproots (Figures 2, 3). The concentrations observed for the P- and K-fertilized plants are in general agreement with P and K concentrations in taproots of well-fertilized alfalfa previously reported by others (Jung and Smith, 1961b; Li et al., 1996; Jungers et al., 2019). Using taproot P and K concentrations from May 2004 and the relationships between taproot and herbage P and K concentrations we previously reported (Berg et al., 2018), herbage P concentrations are estimated to be 1 and 2.5 g kg^−1^ for the 0P and 75P treatments, respectively. Similarly, herbage K concentrations are predicted to be 10 and 25 g kg^−1^ for the 0K and 400K treatments, respectively. These differences in predicted herbage tissue concentrations are consistent with deficient vs. sufficient concentrations reported previously (Lissbrant et al., 2010; Jungers et al., 2019 and references cited therein).

Taproot P and K concentrations changed significantly during cold acclimation and when shoot growth resumed in spring but, with the exception of taproot P in spring (Figure 2), these changes were modest when compared to fertilizer-induced differences in taproot P and K. Similar declines in taproot P when spring growth resumed were reported previously for well-fertilized alfalfa (Jung and Smith, 1961b). We also previously reported reductions in P concentrations of taproots of well-fertilized alfalfa when growth resumed in spring, changes that were associated, in part, with depletion of taproot phytate (Li et al., 1996). In the current study, RNA accumulated in taproots in late fall and the decline in spring when growth resumed (Figure 8). This suggests that the taproot phytate and RNA pools may serve as a source of P for regrowing shoots in spring when P uptake from soil cannot meet the P needs of shoot growth (Jonasson and Chapin, 1991; Oyarzabal and Oesterheld, 2009). Ribosomal RNA contains approximately 50% of total cell P in vegetative tissues of plants, which is a far greater proportion than P accumulated in the inorganic- and phytate-P pools in alfalfa taproots (Campbell et al., 1991; Raven, 2012). In addition, specific RNAases are induced under P-limited conditions that are thought to liberate P from RNA that is subsequently mobilized to growing tissues (Bariola et al., 1994; Smith et al., 2018). Because we only quantified RNA in Year 2, additional research is needed to clarify the taproot P pools (inorganic P, phytate, RNA, and others) involved in providing P to regrowing alfalfa shoots.

Accumulation of taproot C and N reserves during cold acclimation in fall is critical for winter survival and initial growth in spring of perennial legumes (Bula and Smith, 1954; Ruelke and Smith, 1956; Jung and Smith, 1961a; Li et al., 1996; Cunningham and Volenec, 1998; Haagenson et al., 2003a). Little is known regarding the impact of P and K nutrition on taproot C and N reserve accumulation patterns in fall and subsequent reserve use in spring. Trends in taproot starch loss and re-accumulation following defoliation September 7 were similar for all fertility treatments (Figure 5) and typical of previous reports with well-fertilized alfalfa (Bula and Smith, 1954; Jung and Smith, 1961a; Li et al., 1996). However, K-sufficient plants maintained higher taproot starch concentrations throughout fall. This contrasts a recent report where 2 years of K fertilization had no effect on taproot starch concentrations of plants sampled in November in Georgia, United States (Thinguldstad et al., 2020). By comparison, when sampled in summer, P-deficient plants generally had higher taproot starch concentrations irrespective of K fertilizer application (Berg et al., 2009). These authors suggested that P deficiency may have limited C export from taproot amyloplasts impairing both shoot regrowth and taproot starch use after defoliation. In this study, the higher starch in taproots of K-fertilized plants may result from stimulated activities of starch synthase and ADPglucose pyrophosphorylase, key enzymes in starch synthesis whose activities are enhanced by K (Nitros and Evans, 1969; Hawker et al., 1979). As previously reported (Bula and Smith, 1954; Li et al., 1996), taproot starch concentrations declined markedly between December and March, a response attributed in large part to respiratory losses (Avice et al., 1996). Despite this large reduction, the relative ranking of fertilizer treatments remained consistent between December and March. Taproot starch accumulated from March to May for all fertilizer treatments and was slightly higher in the 400K-0P treatment at some samplings. Final taproot starch concentrations in May were approximately 200 g/kg dry matter, a value that agrees with previous reports for well-fertilized alfalfa (Li et al., 1996). The high forage yield of the 400K-75P treatment in May (Table 1) was not closely associated with the high taproot starch concentrations when growth resumed in late March (Figure 5), but taproots of these plants did have the highest taproot protein concentrations of all treatments at this time (Figure 6). Previous work (Boyce and Volenec, 1992) also showed little association between taproot starch concentrations and alfalfa shoot growth among genotypes that differed two-fold in taproot starch concentrations. The more rapid shoot regrowth rate of the low-starch genotype, compared to the high-starch genotype, continued even when plants were defoliated a second time 14 days after initial herbage removal when taproot starch concentrations of the low starch line were less than 25 g kg^−1^ dry wt. Avice et al. (1997) also reported no relationship between taproot starch concentrations and shoot regrowth rates; instead, as in this study, shoot growth rates were closely associated with taproot protein concentrations. Using ^13^C and ^15^N pulse-chase labeling of taproot organic reserve pools, these authors showed that the depletion of taproot C pools during shoot regrowth primarily supported taproot dark respiration, whereas taproot N pools were mobilized to shoots to meet their N needs (Avice et al., 1997).

Taproot sugar concentrations of K-sufficient plants were lower at the onset of the study when compared to K-deficient plants (Figure 4). Defoliation on September 7 reduced taproot sugar concentration irrespective of fertilizer treatment. This was followed by rapid sugar re-accumulation by Day 28 during which time fertility-related differences present initially were maintained. We previously reported low sugar concentrations in taproots of K-fertilized plants when sampled at defoliation in June that were sustained throughout the 30-d regrowth period (Berg et al., 2009). Irrespective of fertility, taproot sugar concentrations averaged approximately 175 g/kg on December 7; a value that agrees with previous reports for well-fertilized alfalfa sampled in December (Jung and Smith, 1961a; Li et al., 1996; Berg et al., 2018). By comparison, K fertilization had no impact on sugar concentrations of taproots sampled in early November in Coastal Plains soils of Georgia, United States (Thinguldstad et al., 2020). Previous work with alfalfa cultivars differing in fall dormancy and winter hardiness indicated minimal winter injury occurred when taproot sugar concentrations were 140 g/kg or greater (Cunningham et al., 2001). This critical value is supported by this study since P and K fertilizer had modest impact on plant persistence (Table 1) and taproots of all fertilizer treatments were above this critical sugar concentration in December (Figure 4).

Taproot sugar concentrations declined for all fertilizer treatments between December 7 and March 27 when shoot growth resumed (Figure 4). Unlike fall taproot samplings, the P-sufficient plants (not the K-sufficient) had lower taproot sugar concentrations at the initial samplings in spring irrespective of K fertilizer application. The reason for this change from December to March is unknown, but we speculate that high taproot P concentrations of P-sufficient plants and its mobilization to shoots (Figure 2) may have stimulated early shoot growth that also led to a greater use of taproot sugars for shoot growth and respiration. While we did not measure early season shoot mass, forage yield in May was ultimately greatest for the P-fertilized treatments (Table 1). Additional work is needed to determine the potential interaction of taproot P and sugar mobilization during early spring growth of alfalfa. Sugar concentrations at forage harvest on May 25 were lowest in taproots of K-fertilized plants agreeing with what was observed the preceding September. As with taproot starch, there was no clear association between taproot sugar concentrations at harvest on Day 49 and the higher forage yield of the 400K-75P fertilizer treatment.

In contrast to taproot C reserves, concentrations of protein and amino-N were generally greater in taproots of the 400K-75P fertilizer treatment and positively associated with its consistently higher herbage yield in May (Figures 6, 7; Table 1). In addition, plants in the 0K-0P fertility treatment had the lowest concentrations of these N pools during spring growth and yielded only 43% that of the 400K-0P fertility treatment. Previous research revealed that K fertilization stimulated N_2_ fixation of alfalfa several fold over rates observed for K-deficient plants (Collins and Duke, 1981). Little change in taproot protein concentration occurred after plants were defoliated September 7. This agrees with previous results (Hendershot and Volenec, 1992), whereas Haagenson et al. (2003b) observed lower protein concentrations in taproots sampled 1 month after a September defoliation. Defoliation in summer generally reduces protein concentrations in taproots sampled 10 to 20 days post-defoliation (Hendershot and Volenec, 1993; Berg et al., 2009), and ^15^N labeling verified transfer of N from taproots to regrowing shoots (Avice et al., 1996; Barber et al., 1996). The extensive accumulation of protein in taproots in fall between Days 28 and 84 is consistent with previous findings with well-fertilized alfalfa (Jung and Smith, 1961a; Li et al., 1996), and while this trend was apparent for all fertilizer treatments, concentrations were greater in the high-yielding 400K-75P treatment.

Taproot protein concentrations declined between December 7 and March 27 of the following year (Figure 6). Some of this reduction in taproot protein may reflect transfer of protein-N from taproots to the very small shoots on crowns present at the initial sampling in spring. However, this reduction in protein also coincided with increased amino-N concentrations taproots in the March 27 sampling (Figure 7). Taproot protein concentrations of all fertility treatments declined until Day 14, then increased after Day 28 presumably as rates of N_2_ fixation increased, and met the N needs of growing shoots with excess N restored in taproots. Previous findings indicate that N_2_ fixation rate is initially very low in spring, and increases markedly as plants enter the late vegetative/bud growth stages (Wivstad et al., 1987). Using ^15^N labeling, Kelner et al. (1997) confirmed that first-harvest herbage derived only 47% of its N from N_2_ fixation, whereas over 80% of herbage N is derived from N_2_ fixation at later harvests. This lower contribution of N_2_ fixation for first-harvest herbage N would be expected if taproot N (and soil N) pools were contributing N to regrowing shoots. Protein concentrations at harvest on May 25 were greater in taproots of K-fertilized plants when compared to K-deficient plants. This agrees with Berg et al. (2018) who reported low protein concentrations in taproots of K-deficient plants when compared to the K-sufficient plants. Thus, adequate K nutrition increases taproot protein concentrations that are important N sources for initial shoot growth in the spring.

Taproots of plants provided K generally contained higher concentrations of amino-N in fall when compared to taproots of plants not fertilized with K (Figure 7). Berg et al. (2018) also observed low taproot amino-N concentrations in K-deficient alfalfa when sampled in December. Defoliation on early September reduced taproot amino-N concentrations of all fertility treatments as previously reported for well-fertilized alfalfa defoliated in summer (Hendershot and Volenec, 1993; Berg et al., 2009). This net loss of taproot amino-N suggests it is a component of the N pool transferred from taproots to shoots during early regrowth previously demonstrated using ^15^N-labeling (Avice et al., 1996; Barber et al., 1996). While all fertilizer treatments accumulated amino-N in taproots in fall between Days 28 and 70, the final concentrations in taproots of the 0K-0P treatment were less than the other treatments on December 7. Jung and Smith (1961a) reported a doubling of soluble, non-protein N pool in taproots of well-fertilized alfalfa and red clover between mid-August and early November. Accumulation of soluble, non-protein N was positively associated with reduced freezing injury measured as electrolyte leakage from taproots of both species.

In general, little change in taproot amino-N occurred overwinter, but a nearly two-fold range in amino-N was observed at the first sampling March 27 (Figure 7). As observed with taproot protein (Figure 6), amino-N declined for all treatments until Day 21 as shoot growth began in spring, then increased from Days 28 to 49. This increase likely reflects the increase in N_2_ fixation during late vegetative development that meets shoot N needs and excess N is partitioned to taproots (Wivstad et al., 1987). As with taproot protein pools, there is a positive association between herbage yield in May (Table 1) and taproot amino-N concentrations in spring.

## Conclusion

Fertilization with contrasting rates of P and K resulted in the expected differences in P and K concentrations in taproot tissues of alfalfa, and these often varied as plants hardened for winter between September and December and/or when shoot growth resumed the following spring. Altered P and K nutrition resulted in large differences in taproot sugar, starch, protein, amino-N and RNA concentrations, and concentrations of these C, N, and P pools generally increased between mid-September and early December as plants hardened, and declined when shoot growth resumed in March. The larger shoots and higher herbage yield of the 400-K-75P plants harvested in May were associated with high concentrations of protein, amino-N, and RNA pools in December and extensive use when growth resumed the following March when compared to plants not fertilized with P and K.

## Data Availability Statement

The raw data supporting the conclusions of this article will be made available by the authors, without undue reservation.

## Author Contributions

WB, SB, SC, and JV designed the experiment. WB, SC, and JV collected the samples in the field, and WB and SC conducted the laboratory analyses. WB, SB, and JV analyzed the data, interpreted the results, and wrote the manuscript. All authors reviewed and edited the manuscript.

## Conflict of Interest

The authors declare that the research was conducted in the absence of any commercial or financial relationships that could be construed as a potential conflict of interest.

## Publisher’s Note

All claims expressed in this article are solely those of the authors and do not necessarily represent those of their affiliated organizations, or those of the publisher, the editors and the reviewers. Any product that may be evaluated in this article, or claim that may be made by its manufacturer, is not guaranteed or endorsed by the publisher.

## References

[ref1] Avice J. C. Ourry A. Lemaire G. Boucaud J. (1996). Nitrogen and carbon flows estimated by ^15^N and ^13^C pulse-chase labeling during regrowth of alfalfa. Plant Physiol. 112, 281–290. 10.1104/pp.112.1.281, PMID: 12226391 PMC157947

[ref2] Avice J. C. Ourry A. Lemaire G. Volenec J. J. Boucaud J. (1997). Root protein and vegetative storage protein are key organic nutrients for alfalfa shoot regrowth. Crop Sci. 37, 1187–1193. 10.2135/cropsci1997.0011183X003700040027x

[ref3] Barber L. D. Joern B. C. Volenec J. J. Cunningham S. M. (1996). Supplemental nitrogen effects on alfalfa regrowth and nitrogen mobilization from roots. Crop Sci. 36, 1217–1223. 10.2135/cropsci1996.0011183X003600050025x

[ref4] Bariola P. A. Howard C. J. Taylor C. B. Verburg M. T. Jaglan V. D. Green P. J. (1994). The *Arabidopsis* ribonuclease gene RNS1 is tightly controlled in response to phosphate limitation. Plant J. 6, 673–685. 10.1046/j.1365-313X.1994.6050673.x, PMID: 8000425

[ref5] Barta A. L. (1982). Response of symbiotic N_2_ fixation and assimilate partitioning to K supply in alfalfa. Crop Sci. 22, 89–92. 10.2135/cropsci1982.0011183X002200010020x

[ref6] Berg W. K. Cunningham S. M. Brouder S. M. Joern B. C. Johnson K. D. Volenec J. J. (2009). Influence of phosphorus and potassium on alfalfa yield, taproot C and N pools, and transcript levels of key genes after defoliation. Crop Sci. 49, 974–982. 10.2135/cropsci2008.07.0395

[ref7] Berg W. K. Cunningham S. M. Brouder S. M. Johnson K. D. Joern B. C. Volenec J. J. (2005). Influence of phosphorus and potassium fertilization on alfalfa yield and yield components. Crop Sci. 45, 297–304. 10.2135/cropsci2005.0297

[ref8] Berg W. K. Cunningham S. M. Brouder S. M. Johnson K. D. Joern B. C. Volenec J. J. (2007). The long-term impact of phosphorus and potassium fertilization on alfalfa yield and yield components. Crop Sci. 47, 2198–2209. 10.2135/cropsci2006.09.0576

[ref9] Berg W. K. Lissbrant S. Cunningham S. M. Brouder S. M. Volenec J. J. (2018). Phosphorus and potassium effects on taproot C and N reserve pools and long-term persistence of alfalfa (*Medicago sativa* L.). Plant Sci. 272, 301–308. 10.1016/j.plantsci.2018.02.026, PMID: 29807603

[ref10] Boyce P. J. Volenec J. J. (1992). Taproot carbohydrate concentrations and stress tolerance of alfalfa. Crop Sci. 32, 757–761. 10.2135/cropsci1992.0011183X003200030036x

[ref11] Bradford M. M. (1976). A rapid and sensitive method for the quantification of microgram quantities of protein utilizing the principle of protein dye binding. Anal. Biochem. 72, 248–254. 10.1016/0003-2697(76)90527-3, PMID: 942051

[ref12] Bray R. H. Kurtz L. T. (1945). Determination of total, organic, and available forms of phosphorus in soils. Soil Sci. 59, 39–45. 10.1097/00010694-194501000-00006

[ref13] Brown B. A. (1928). Effect of fertilizers on maintaining stands of alfalfa. Agron. J. 20, 109–117. 10.2134/agronj1928.00021962002000020003x

[ref14] Bula R. J. Smith D. (1954). Cold resistance and chemical composition in overwintering alfalfa, red clover, and sweetclover. Agron. J. 46, 397–401. 10.2134/agronj1954.00021962004600090001x

[ref15] Bula R. J. Smith D. Hodgson H. J. (1956). Cold resistance in alfalfa from two diverse latitudes. Agron. J. 48, 153–156. 10.2134/agronj1956.00021962004800040002x

[ref16] Campbell M. Dunn R. Ditterline R. Pickett S. Raboy V. (1991). Phytic acid represents 10 to 15% of total phosphorus in alfalfa root and crown. J. Plant Nutr. 14, 925–937. 10.1080/01904169109364253

[ref17] Castonguay Y. Laberge S. Brummer E. C. Volenec J. J. (2006). Alfalfa winter hardiness: a research retrospective and integrated perspective. Adv. Agron. 90, 203–265. 10.1016/S0065-2113(06)90006-6

[ref18] Collins M. Duke S. H. (1981). Influence of potassium-fertilization rate and form on photosynthesis and N_2_ fixation of alfalfa. Crop Sci. 21, 481–485. 10.2135/cropsci1981.0011183X002100040001x

[ref19] Collins M. Lang D. J. Kelling K. A. (1986). Effects of phosphorus, potassium and sulfur on alfalfa nitrogen-fixation under field conditions. Agron. J. 78, 959–963. 10.2134/agronj1986.00021962007800060005x

[ref20] Conti T. R. Geiger D. R. (1982). Potassium nutrition and translocation in sugar beet. Plant Physiol. 70, 168–172. 10.1104/pp.70.1.168, PMID: 16662439 PMC1067106

[ref21] Cooper R. B. Blaser R. E. Brown R. H. (1967). Potassium nutrition effects on net photosynthesis and morphology of alfalfa. Soil Sci. Soc. Am. Proc. 31, 231–234. 10.2136/sssaj1967.03615995003100020026x

[ref22] Culman S. Fulford A. Camberato J. Steinke K. (2020). Tri-state fertilizer recommendations for corn, soybeans, wheat & alfalfa. The Ohio State University, 56. Available at: https://agcrops.osu.edu/FertilityResources/tri-state_info#:~:text=The%20Tri-State%20Fertilizer%20Recommendations%20for%20Corn%2C%20Soybeans%2C%20Wheat%2C,manage%20nutrients%20as%20judiciously%20and%20profitably%20as%20possible (Accessed July 6, 2021).

[ref23] Cunningham S. M. Gana J. A. Volenec J. J. Teuber L. R. (2001). Winter hardiness, root physiology, and gene expression in successive fall dormancy selections from ‘Mesilla’ and ‘CUF 101’ alfalfa. Crop Sci. 41, 1091–1098. 10.2135/cropsci2001.4141091x

[ref24] Cunningham S. M. Nadeau P. Castonguay Y. Leberge S. Volenec J. J. (2003). Raffinose and stachyose accumulation, galactinol synthase expression, and winter injury of contrasting *Medicago sativa* germplasms. Crop Sci. 43, 562–570. 10.2135/cropsci2003.0562

[ref25] Cunningham S. M. Volenec J. J. (1996). Purification and characterization of vegetative storage proteins from alfalfa (*Medicago sativa* L.). J. Plant Physiol. 147, 625–632. 10.1016/S0176-1617(11)81469-0

[ref26] Cunningham S. M. Volenec J. J. (1998). Seasonal carbohydrate and protein metabolism in roots of contrasting alfalfa (*Medicago sativa* L.) cultivars. J. Plant Physiol. 153, 220–225. 10.1016/S0176-1617(98)80069-2

[ref27] Dietz K. J. Foyer C. (1986). The relationship between phosphate status and photosynthesis in leaves. Reversibility of the effects of phosphate deficiency on photosynthesis. Planta 167, 376–381. 10.1007/BF00391342, PMID: 24240307

[ref28] Duke S. H. Collins M. Soberalske R. M. (1980). Effects of potassium fertilization on nitrogen fixation and nodule enzymes of nitrogen metabolism in alfalfa. Crop Sci. 20, 213–219. 10.2135/cropsci1980.0011183X002000020016x

[ref29] Elliott A. C. Woodward W. A. (2016). SAS Essentials. Mastering SAS for Data Analytics. 2nd *Edn*. Hoboken, NJ, USA: John Wiley and Sons, 338–346.

[ref30] Escalada J. A. Smith D. (1972). Changes in nonstructural carbohydrate fractions at intervals down the tap root bark and wood of alfalfa (*Medicago sativa* L.) during regrowth. Crop Sci. 12, 745–749. 10.2135/cropsci1972.0011183X001200060008x

[ref31] Foyer C. Spencer C. (1986). The relationship between phosphate status and photosynthesis in leaves. Effects on intracellular orthophosphate distribution, photosynthesis and assimilate partitioning. Planta 167, 369–375. 10.1007/BF00391341, PMID: 24240306

[ref32] Graber L. F. Nelson N. T. Luekel W. A. Albert W. B. (1927). Organic food reserves in relation to the growth of alfalfa and other perennial herbaceous plants. Res. Bull. 80, 1–128.

[ref33] Haagenson D. M. Cunningham S. M. Volenec J. J. (2003a). Root physiology of less fall dormant, winter hardy alfalfa selections. Crop Sci. 43, 1441–1447. 10.2135/cropsci2003.1441

[ref34] Haagenson D. M. Cunningham S. M. Joern B. C. Volenec J. J. (2003b). Autumn defoliation effects on alfalfa winter survival, root physiology, and gene expression. Crop Sci. 43, 1340–1348. 10.2135/cropsci2003.1340

[ref35] Hart A. L. Greer D. H. (1988). Photosynthesis and carbon export in white clover plants grown at various levels of phosphorus supply. Physiol. Plant. 73, 46–51. 10.1111/j.1399-3054.1988.tb09191.x

[ref36] Hawker J. S. Marschner H. Krauss A. (1979). Starch synthesis in developing potato tubers. Physiol. Plant. 46, 25–30. 10.1111/j.1399-3054.1979.tb03180.x

[ref37] Hendershot K. L. Volenec J. J. (1992). Taproot nitrogen accumulation and use in overwintering alfalfa (*Medicago sativa* L.). J. Plant Physiol. 141, 68–74. 10.1016/S0176-1617(11)80853-9

[ref38] Hendershot K. L. Volenec J. J. (1993). Nitrogen pools in taproots of *Medicago sativa* L. after defoliation. J. Plant Physiol. 141, 129–135. 10.1016/S0176-1617(11)80748-0

[ref39] Hu W. Ren T. Meng F. Cong R. Li X. White P. J.. (2019). Leaf photosynthetic capacity is regulated by the interaction of nitrogen and potassium through coordination of CO_2_ diffusion and carboxylation. Physiol. Plant. 167, 418–432. 10.1111/ppl.12919, PMID: 30690727

[ref40] Jonasson S. Chapin F. S. (1991). Seasonal uptake and allocation of phosphorus in *Eriophorum vaginatum* L measured by labelling with ^32^P. New Phytol. 118, 349–357. 10.1111/j.1469-8137.1991.tb00987.x, PMID: 33874176

[ref41] Jung G. A. Smith D. (1959). Influence of soil potassium and phosphorus content on the cold resistance of alfalfa. Agron. J. 51, 585–587. 10.2134/agronj1959.00021962005100100004x

[ref42] Jung G. A. Smith D. (1961a). Trends of cold resistance and chemical changes over winter in the roots and crowns of alfalfa and medium red clover. I. Changes in certain nitrogen and carbohydrate fractions. Agron. J. 53, 359–364. 10.2134/agronj1961.00021962005300060001x

[ref43] Jung G. A. Smith D. (1961b). Trends of cold resistance and chemical changes over winter in the roots and crowns of alfalfa and medium red clover II. Changes in certain mineral constituents. Agron. J. 53, 364–366. 10.2134/agronj1961.00021962005300060002x

[ref44] Jungers J. M. Kaiser D. E. Lamb J. F. S. Lamb J. A. Noland R. L. Samac D. A.. (2019). Potassium fertilization affects alfalfa forage yield, nutritive value, root traits, and persistence. Agron. J. 111, 1–10. 10.2134/agronj2019.01.0011

[ref45] Kelner D. J. Vessey J. K. Entz M. H. (1997). The nitrogen dynamics of 1-, 2-and 3-year stands of alfalfa in a cropping system. Agric. Ecosyst. Environ. 64, 1–10. 10.1016/S0167-8809(97)00019-4

[ref46] Kitchen N. R. Buchholz D. D. Nelson C. J. (1990). Potassium fertilizer and potato leafhopper effects on alfalfa growth. Agron. J. 82, 1069–1074. 10.2134/agronj1990.00021962008200060008x

[ref47] Li R. Volenec J. J. Joern B. C. Cunningham S. M. (1996). Seasonal changes in nonstructural carbohydrates, protein, and macronutrients in roots of alfalfa, red clover, sweetclover, and birdsfoot trefoil. Crop Sci. 36, 617–623. 10.2135/cropsci1996.0011183X003600030016x

[ref48] Li R. Volenec J. J. Joern B. C. Cunningham S. M. (1997). Potassium and nitrogen effects on carbohydrate and protein metabolism in alfalfa roots. J. Plant Nutr. 20, 511–529. 10.1080/01904169709365271

[ref49] Li R. Volenec J. J. Joern B. C. Cunningham S. M. (1998). Effects of phosphorus nutrition on carbohydrate and protein metabolism in alfalfa roots. J. Plant Nutr. 21, 459–474. 10.1080/01904169809365417

[ref50] Lissbrant S. Brouder S. M. Cunningham S. M. Volenec J. J. (2010). Identification of fertility regimes that enhance long-term productivity of alfalfa using cluster analysis. Agron. J. 102, 580–591. 10.2134/agronj2009.0300

[ref51] Lloveras J. Chocarro C. Torres L. Viladrich D. Costafreda R. Santiveri F. (2012). Alfalfa yield components and soil potassium depletion as affected by potassium fertilization. Agron. J. 104, 729–734. 10.2134/agronj2011.0293

[ref52] Lu X. Ji S. Hou C. Qu H. Li P. Shen Y. (2018). Impact of root C and N reserves on shoot regrowth of defoliated alfalfa cultivars differing in fall dormancy. Grassl. Sci. 64, 83–90. 10.1111/grs.12190

[ref53] Mitchell M. L. Clark S. G. Butler K. L. Zhongnan N. Burnett V. F. Meyer R.. (2020). Harvest interval affects lucerne (*Medicago sativa* L.) taproot total yield, starch, nitrogen, and water-soluble carbohydrates. J. Agron. Crop Sci. 206, 619–629. 10.1111/jac.12397

[ref54] Nelson D. W. Sommers L. E. (1973). Determination of total nitrogen in plant material. Agron. J. 65, 109–112. 10.2134/agronj1973.00021962006500010033x

[ref55] Nitros R. E. Evans H. J. (1969). Effects of univalent cations on the activity of particulate starch synthetase. Plant Physiol. 44, 1260–1266. 10.1104/pp.44.9.1260, PMID: 16657200 PMC396253

[ref56] Ougham H. J. Davis T. G. E. (1990). Leaf development in *Lolium temulentum*. Gradients of RNA complement and plastid and non-plastid transcripts. Physiol. Plant. 79, 331–338. 10.1111/j.1399-3054.1990.tb06750.x

[ref57] Oyarzabal M. Oesterheld M. (2009). Phosphorus reserves increase grass regrowth after defoliation. Oecologia 159, 717–724. 10.1007/s00442-008-1263-z, PMID: 19132398

[ref58] Rao I. M. Fredeen A. L. Terry N. (1990). Leaf phosphate status, photosynthesis, and carbon partitioning in sugarbeet III. Diurnal changes in carbon partitioning and carbon export. Plant Physiol. 92, 29–36. 10.1104/pp.92.1.29, PMID: 16667261 PMC1062243

[ref59] Raven J. A. (2012). Protein turnover and plant RNA and phosphorus requirements in relation to nitrogen fixation. Plant Sci. 188, 25–35. 10.1016/j.plantsci.2012.02.010, PMID: 22525241

[ref60] Roberts J. L. Olson F. R. (1944). Influence of phosphorus and potassium on symbiotic nitrogen fixation. Agron. J. 36, 637–645. 10.2134/agronj1944.00021962003600080001x

[ref61] Rosen H. (1957). A modified ninhydrin colorimetric analysis for amino acids. Arch. Biochem. Biophys. 67, 10–15. 10.1016/0003-9861(57)90241-2, PMID: 13412116

[ref62] Ruelke O. C. Smith D. (1956). Overwintering trends of cold resistance and carbohydrates in medium red, ladino, and common white clover. Plant Physiol. 31, 364–368. 10.1104/pp.31.5.364, PMID: 16654901 PMC540805

[ref63] SAS Institute Inc. (2015). SAS/STAT User’s Guide Release 9.4. Cary, NC, USA: SAS Institute Inc., 440.

[ref64] Sims J. T. (2009). “Soil test phosphorus: principles and methods,” in Methods of Phosphorus Analysis for Soils, Sediments, Residuals, and Waters. 2nd *edn*. eds. Kovar J. L. Pierzynski G. M. (Blacksburg, VA USA: Virginia Tech University), 9–19.

[ref65] Smith A. P. Fontenot E. B. Zahraeifard S. DiTusa S. F. (2018). Molecular components that drive phosphorus-remobilisation during leaf senescence. Annu. Plant Rev. 48, 159–186. 10.1002/9781119312994.apr0521

[ref66] Thinguldstad B. Tucker J. J. Baxter L. L. Segers J. R. Hancock D. W. Stewart R. L. (2020). Alfalfa response to low potassium under different harvest regimes in coastal plains. Agrosyst. Geosci. Environ. 3:e20029. 10.1002/agg2.20029

[ref67] Tighe-Neira R. Alberdi M. Arce-Johnson P. Romero J. Reyes-Díaz M. Rengel Z.. (2018). “Role of potassium in governing photosynthetic processes and plant yield,” in Plant Nutrients and Abiotic Stress Tolerance. eds. Hasanuzzaman M. Fujita M. Oku H. Nahar K. Hawrylak-Nowak B. (Singapore: Springer), 191–203.

[ref68] Van Handel E. (1968). Direct microdetermination of sucrose. Anal. Biochem. 22, 1341–1346. 10.1016/0003-2697(68)90317-5 5641848

[ref69] Volenec J. J. Brouder S. M. Murrell T. S. (2021). “Broadening the objectives of future potassium recommendations,” in Improving Potassium Recommendations for Agricultural Crops. eds. Murrell T. S. Mikkelsen R. L. Sulewski G. Norton R. Thompson M. L. (Cham, Switzerland: Springer Nature), 385–415.

[ref70] Volenec J. J. Ourry A. Joern B. C. (1996). A role for nitrogen reserves in forage regrowth and stress tolerance. Physiol. Plant. 97, 185–193. 10.1111/j.1399-3054.1996.tb00496.x

[ref71] Wang J. Zhu G. Dong Y. Zhang H. Rengel Z. Ai Y.. (2018). Potassium starvation affects biomass partitioning and sink–source responses in three sweet potato genotypes with contrasting potassium-use efficiency. Crop Pasture Sci. 69, 506–514. 10.1071/CP17328

[ref72] Wivstad M. Mårtensson A. M. Ljunggren H. D. (1987). Field measurement of symbiotic nitrogen fixation in an established lucerne ley using ^15^N and an acetylene reduction method. Plant Soil 97, 93–104. 10.1007/BF02149828

[ref73] Wolf D. D. Kimbrough E. L. Blaser R. E. (1976). Photosynthetic efficiency of alfalfa with increasing potassium nutrition. Crop Sci. 16, 292–294. 10.2135/cropsci1976.0011183X001600020035x

